# Variation in Complexity of Infection and Transmission Stability between Neighbouring Populations of *Plasmodium vivax* in Southern Ethiopia

**DOI:** 10.1371/journal.pone.0140780

**Published:** 2015-10-15

**Authors:** Sisay Getachew, Sheren To, Hidayat Trimarsanto, Kamala Thriemer, Taane G. Clark, Beyene Petros, Abraham Aseffa, Ric N. Price, Sarah Auburn

**Affiliations:** 1 College of Natural Sciences, Addis Ababa University, P.O. Box 52, Addis Ababa, Ethiopia; 2 Armauer Hansen Research Institute, P.O. Box 1005, Jimma Road, Addis Ababa, Ethiopia; 3 Global and Tropical Health Division, Menzies School of Health Research and Charles Darwin University, Darwin, NT 0810, Australia; 4 Eijkman Institute for Molecular Biology, Jl. Diponegoro 69, Jakarta Pusat 10430, Indonesia and The Ministry of Research and Technology (RISTEK), Jakarta, Indonesia; 5 Agency for Assessment and Application of Technology, Jl. MH Thamrin 8, Jakarta 10340, Indonesia; 6 Faculty of Infectious and Tropical Diseases, London School of Hygiene and Tropical Medicine, Keppel Street, London, WC1E 7HT, United Kingdom; 7 Faculty of Epidemiology and Population Health, London School of Hygiene and Tropical Medicine, Keppel Street, London, WC1E 7HT, United Kingdom; 8 Centre for Tropical Medicine and Global Health, Nuffield Department of Clinical Medicine, University of Oxford, Oxford, United Kingdom; Walter & Eliza Hall Institute, AUSTRALIA

## Abstract

**Background:**

*P*. *vivax* is an important public health burden in Ethiopia, accounting for almost half of all malaria cases. Owing to heterogeneous transmission across the country, a stronger evidence base on local transmission dynamics is needed to optimise allocation of resources and improve malaria interventions.

**Methodology and Principal Findings:**

In a pilot evaluation of local level *P*. *vivax* molecular surveillance in southern Ethiopia, the diversity and population structure of isolates collected between May and November 2013 were investigated. Blood samples were collected from microscopy positive *P*. *vivax* patients recruited to clinical and cross-sectional surveys from four sites: Arbaminch, Halaba, Badawacho and Hawassa. Parasite genotyping was undertaken at nine tandem repeat markers. Eight loci were successfully genotyped in 197 samples (between 36 and 59 per site). Heterogeneity was observed in parasite diversity and structure amongst the sites. Badawacho displayed evidence of unstable transmission, with clusters of identical clonal infections. Linkage disequilibrium in Badawacho was higher (*I*_AS_ = 0.32, *P* = 0.010) than in the other populations (*I*_AS_ range = 0.01–0.02) and declined markedly after adjusting for identical infections (*I*_AS_ = 0.06, *P* = 0.010). Other than Badawacho (*H*_E_ = 0.70), population diversity was equivalently high across the sites (*H*_E_ = 0.83). Polyclonal infections were more frequent in Hawassa (67%) than the other populations (range: 8–44%). Despite the variable diversity, differentiation between the sites was low (*F*_ST_ range: 5 x 10^−3^–0.03).

**Conclusions:**

Marked variation in parasite population structure likely reflects differing local transmission dynamics. Parasite genotyping in these heterogeneous settings has potential to provide important complementary information with which to optimise malaria control interventions.

## Introduction

Once considered a relatively benign infection, *Plasmodium vivax* is now acknowledged to be an important public health threat capable of causing severe and fatal disease [1–3]. Whilst the greatest burden of infection is in South and Southeast Asia, the Horn of Africa harbours a considerable proportion of the global reservoir of infections, and an estimated 10–20% of all *P*. *vivax* cases [4]. The greatest number of clinical cases is reported from Ethiopia, where malaria continues to be a major cause of morbidity and mortality, accounting for almost 15% of all outpatient visits and 10% of hospital admissions [5]. The high proportion of *P*. *vivax* infections is a likely reflection of the large number of Duffy positive individuals [6]. Chloroquine has been the first-line treatment of uncomplicated *P*. *vivax* in Ethiopia for over five decades, however drug resistant parasites are emerging, particularly in the south of the country [7–10]. Containment strategies of *P*. *vivax* are underway, however, relative to *P*. *falciparum*, *P*. *vivax* has proven far harder to control [11, 12]: new approaches are needed to reduce the transmission of this highly adaptive pathogen.

Wide variation in climate, ecology and human settlement patterns across Ethiopia have resulted in extensive heterogeneity in malaria epidemiology. The greatest threat occurs in the 40 million people living between altitudes of 1,500 and 2,500 metres, where unstable transmission coupled with a receptive environment gives rise to sporadic malaria outbreaks [13, 14]. Current epidemiological surveillance strategies aim to identify populations at greatest risk to prioritise malaria control activities. New molecular approaches are being increasingly used to further characterise parasite populations, quantifying parasite diversity and population structure; these data can provide important insights relevant to the national malaria control program [15–38]. A previous study examined the genetic diversity of *P*. *vivax* isolates collected between 2006 and 2008 in Assendabo, Ethiopia, providing broad insights into the population structure and diversity of African versus Asian isolates [20]. However, as the isolates were collected from a single site, investigation of the local variation in parasite diversity and underlying transmission dynamics was not possible.

In an evaluation of the scope of molecular surveillance of *P*. *vivax* transmission dynamics in Ethiopia, genotyping methods were used to characterize the local genetic diversity and structure of isolates collected during the main transmission season in 2013 at four sites in neighbouring zones in the South Nations Nationalities and Peoples’ Region (SNNPR).

## Materials and Methods

### Study Sites and Sample Collection

**Fig 1:**
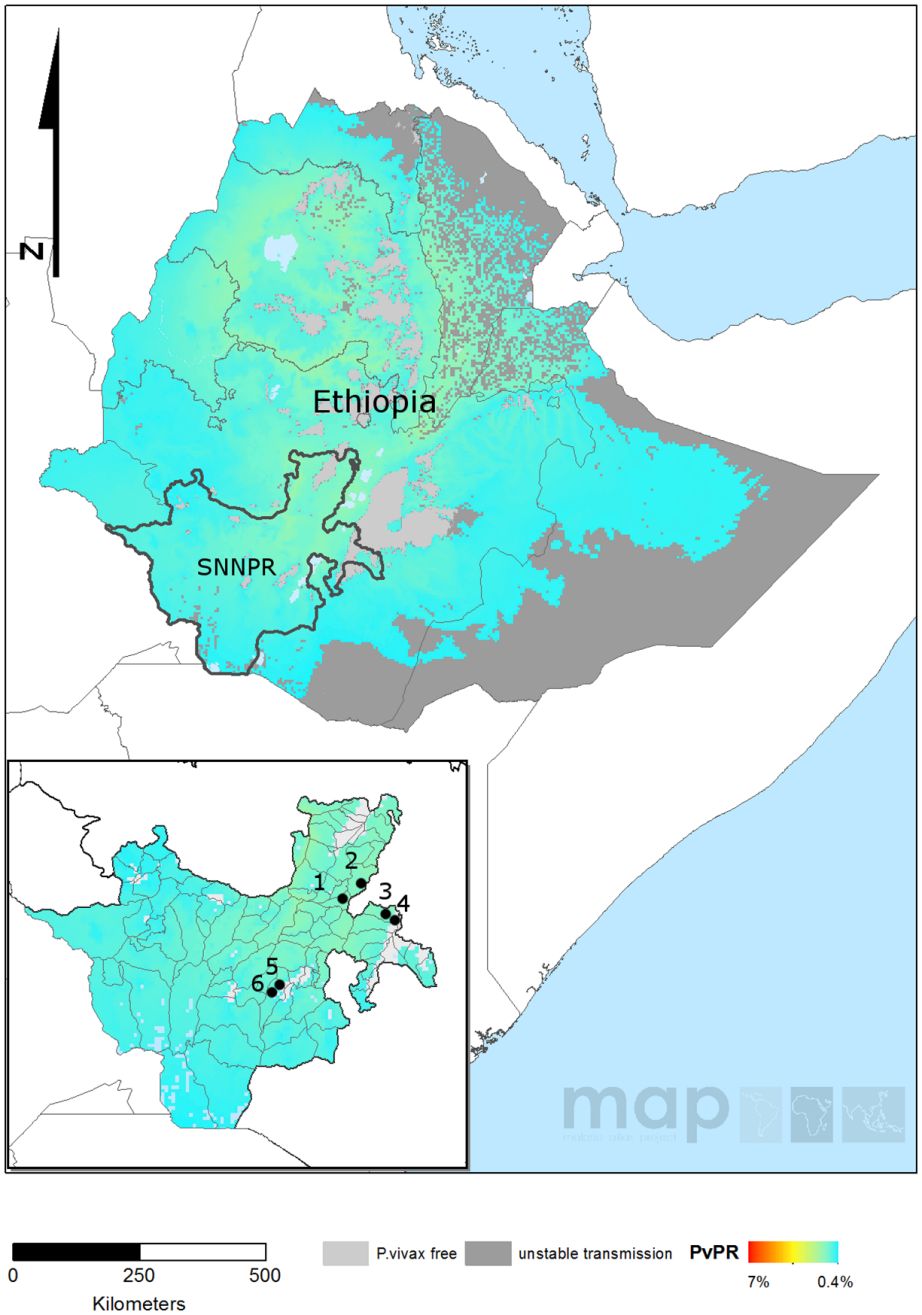
*P*. *vivax* prevalence map for Ethiopia illustrating the location of the study sites. This map was generated by the Malaria Atlas Project, University of Oxford. The colour scale reflects the model-based geostatistical point estimates of the annual mean *P*. *vivax* parasite rate in the 1–99 year age range (*Pv*PR) within the stable spatial limits of *P*. *vivax* transmission in 2010 [39]. The approximate locations of the study sites are indicated with numbered black dots: Shone Health Center, Badawacho (1), Guba Health Center, Halaba (2), Adare Hospital, Hawassa (3), Millenium Health Center, Hawassa (4), Arbaminch Hospital, Arbaminch (5), and Shele Health Center, Arbaminch (6). All MAP maps are available to users under the CCAL 3.0. http://www.map.ox.ac.uk/about-map/open-access/.

The study was conducted in four sites in the SNNPR; Arba Minch (town) and Zuria in Gamo Gofa Zone (Arbaminch), Misrak Badawacho in Hadiya Zone (Badawacho), Halaba Special Woreda (Halaba) and Hawasa town in Sidama Zone (Hawassa) (Fig 1). Details on the local population and malaria epidemiology in each of the districts are provided in the Supplementary Material (S1 Table). Patients were enrolled to the study during the peak malaria transmission season between May and November 2013. In Arbaminch, the majority of patients were enrolled within the framework of a *P*. *vivax* chloroquine sensitivity survey conducted at Shele Health Center. Additional patients were recruited by cross-sectional sampling from Arbaminch Hospital. In Badawacho and Halaba, patients were recruited in the framework of a *P*. *vivax* chloroquine survey conducted at Shone Health Center and Guba Health Center, respectively. In Hawassa, all patients were recruited by cross-sectional sampling at Adare Hospital and Millenium Health Center. Enrolment criteria were uncomplicated *P*. *vivax* mono-infection with microscopy-determined parasite density above 250 μl^-1^, an axillary temperature of ≥37.5°C or history of fever within 48 hours of presentation, and residence in close proximity to the health center (i.e. within 10 km radius). Patients were asked to donate a capillary (50–150 μl) blood sample spotted onto filter paper (Whatman^®^ 3MM filter paper, Cat No.3030917) in addition to a blood sample for routine microscopic examination. Thick and thin blood films were read by two to three independent laboratory technicians from the health center. The number of asexual parasites was counted per 200 white blood cells (WBC) and parasitaemia estimated assuming a WBC count of 8000 μl^-1^.

### Molecular Processing

DNA extraction was undertaken using the QIAamp blood mini kit (Qiagen) according to the manufacturer’s protocol for dried blood spots. Genotyping was undertaken at nine previously described STR markers: *Pv3*.*27*, *msp1F3*, *MS1*, *MS5*, *MS8*, *MS10*, *MS12*, *MS16* and *MS20* [40, 41]. These markers are included in a consensus panel selected by partners within the Vivax Working Group of the Asia Pacific Malaria Elimination Network [42]. The *Pv3*.*27*, *MS16* and *msp1F3* loci were amplified using methods described elsewhere [15]. The protocol for the remaining loci and the details of the primer sequences and chromosomal locations for each marker have been provided previously [15, 20]. The labelled PCR products were sized by denaturing capillary electrophoresis on an ABI 3100 Genetic Analyzer with GeneScan LIZ-600 (Applied Biosystems) internal size standards. Genotype calling was undertaken using GeneMapper Version 4.0. To reduce potential artefacts, an arbitrary fluorescent intensity threshold of 500 rfu was applied for peak detection. All electropherogram traces were additionally inspected manually. For each isolate, at each locus, the predominant allele and any additional alleles with minimum 33% height of the predominant allele were scored [43].

### Population Genetic Analysis

An infection was defined as polyclonal if more than one allele was observed at one or more loci. The multiplicity of infection (MOI) for a given sample was defined as the maximum number of alleles observed at any of the loci investigated. With the exception of measures of polyclonality and MOI, only the predominant allele at each locus in each isolate was used for analysis [43]. The expected heterozygosity (*H*_E_) was measured as an index of population diversity using the formula *H*_E_ = [*n*/ (*n*-1)] [1-Σ*p*_*i*_^2^], where *n* is the number of isolates analyzed and *pi* is the frequency of the *ith* allele in the population. The pairwise *F*_ST_ metric was used to gauge the genetic distance between populations. Calculations were undertaken using Arlequin software (version 3.5) [44]. Standardized measures (*F’*_ST_) were additionally calculated to adjust for high marker diversity [45]. Population structure was further assessed using STRUCTURE software version 2.3.3 [46]. Twenty replicates, with 100,000 burn-in and 100,000 post burn-in iterations were run for each of *K* (populations) from 1–10 using the model parameters of admixture with correlated allele frequencies. The most probable *K* was derived by applying the delta K method [47], and bar plots were prepared with *distruct* software version 1.1 [48]. Multi-locus genotypes (MLGs) were reconstructed from the predominant allele at each locus in isolates with no missing data. Multi-locus linkage disequilibrium (LD) was measured by the standardised index of association (*I*_A_^S^) using the web-based LIAN 3.5 software [49]. The significance of the *I*_A_^S^ estimates was assessed using 10,000 random permutations of the data. For each population, LD was assessed in 1) all samples, 2) samples with a maximum of one multi-allelic locus, and 3) with repeated MLGs represented once. The genetic relatedness between sample pairs was assessed by measuring the proportion of alleles shared between MLG pairs (*ps*). Using (1-*ps*) as a measure of genetic distance [50], an unrooted neighbour-joining tree [51] was generated with the ape package using R software [52]. The correlation between genetic and temporal distance was assessed using Mantel’s *r*-test with 10,000 permutations using the ade4 package in R [53].

### Statistical Analysis

Statistical comparisons of patient gender and infection polyclonality between sites were undertaken using Pearson’s Chi-square test. The significance of difference between sites with regard to patient age, parasite density, MOI and expected heterozygosity were assessed using the Mann-Whitney U test. Assessment of the correlation between MOI with patient age and parasite density was undertaken using Spearman’s rank correlation coefficient. All tests were performed using R software, with a significance threshold of 0.05.

### Ethical Approval

Ethical approval for the study was granted by the respective Ethical Boards of the Addis Ababa University College of Natural Sciences, Ethiopia (RERC/002/05/2013), the Armauer Hansen Research Institute, Addis Ababa, Ethiopia (AHRI-ALERT P011/10), the National Research Ethics Review Committee of Ethiopia (Ref.no. 3.10/580/06) and the Human Research Ethics Committee of the Northern Territory Department of Health and Menzies School of Health Research, Australia (HREC-13-1942). Written informed consent was obtained from all study participants or a parent or guardian where participants were 18 years of age or younger.

## Results

### Patient Sampling

Between May and November 2013, a total of 353 participants with microscopy-positive *P*. *vivax* infection were enrolled in cross-sectional and clinical surveys at multiple health centres and hospitals across the four sites investigated (Table 1). In Arbaminch and Hawassa sites, participants were recruited from two health care facilities in each district; isolates were pooled according to district for analysis. Comparison between Arbaminch Hospital and Shele Health Center did not reveal any significant differences in sample diversity (S2 Table). Owing to the small number of samples from Adare Hospital that were included in the analysis (*n* = 5), comparisons with Millenium Hospital were not undertaken.

**Table 1:** Details of parasite sampling.

Site	Health Facility	Sampling framework	Collection period	No. participants ^1^	Median age of participants (IQR), years	No. male participants	Median parasite density (IQR), ul^-1^	No. samples analyzed ^2^
**Arbaminch**	Arbaminch Hospital	Cross-sectional	Jun-Aug 2013	19	20 (14.5–22.5)	12 (63.2%)	3,213 (2,476–3,420)	15
	Shele Health Center	Clinical survey	May-Nov 2013	89	10 (4–18)	58 (65.2%)	3,520 (1,520–7,200)	21
	Both sites	Cross-sectional and clinical	May-Nov 2013	108	13.5 (5–19.3)	70 (64.8%)	3,247 (1,660–6,705)	36
**Halaba**	Guba Health Center	Clinical survey	Jun-Nov 2013	52	12 (4.8–21.3)	30 (57.7%)	7,698 (2,774–14,450)	47
**Badawacho**	Shone Health Center	Clinical survey	May-Aug 2013	90	5.8 (4–8)	51 (56.7%)	1,172 (800–3,342)	59
**Hawassa**	Adare Hospital	Cross-sectional	Sep 2013	19	25 (21–30)	11 (57.9%)	1,890 (1,236–2,679)	5
	Millenium Health Center	Cross-sectional	Aug-Nov 2013	84	18 (11.8–24.3)	46 (54.8%)	2,312 (1,219–4,544)	50
	Both sites	Cross-sectional	Aug-Nov 2013	103	20 (13–26)	57 (55.3%)	2,300 (1,222–3,780)	55
**All sites**	**All sites**	**Cross-sectional and clinical**	**May-Nov 2013**	**353**	**13 (5–20)**	**207 (58.6%)**	**2,408 (1,200–6.020)**	197

^1^ Number of microscopy-determined *P*. *vivax* positive patients enrolled in the study.

^2^ Number of samples included in the final data set.

The median age of participants was lowest in Badawacho (5.8 years), followed by Halaba (12 years), Arbaminch (13.5 years) and Hawassa (20 years); Table 1. With the exception of Arbaminch versus Halaba (*P* = 0.887), all pairwise comparisons of age were significant (*P* < 0.05). Within both the Arbaminch and Hawassa sites, participants were significantly older at the Hospitals compared to the outpatient clinics (*P* = 3.3 x 10^−4^ and *P* = 0.003 respectively; Table 1). Significant differences were also observed in peripheral parasitaemia, with the lowest median density observed in Badawacho (1,172 ul^-1^), followed by Hawassa (2,300 ul^-1^), Arbaminch (3,247 ul^-1^) and Halaba (7,698 ul^-1^); *P* < 0.05 in all pairwise comparisons. No significant differences were observed in the proportion of male participants, which ranged from 55–66% across the sites.

Filter spot blood samples were available from 61.5% (217) of the 353 *P*. *vivax* microscopy-positive participants, on which parasite genotyping could be attempted. Owing to artefact peaks that were difficult to distinguish from authentic allele peaks, the MS8 locus was excluded from analysis. The remaining 8 loci performed well with 0 (0%) to 10 (5%) genotyping failures across the 217 samples. All 8 markers exhibited moderate to high diversity in each of the four sites (see Supplementary Material; S3 Table). Although MS16 has exhibited apparent excess diversity in other sites [54], we did not observe evidence of excess diversity at this marker in southern Ethiopia, where it fell fifth in order of highest to lowest diversity amongst the 8 loci assessed (S3 Table). Indeed, whilst the diversity at MS16 was moderately high in our study sites (*H*_E_ = 0.85), a previous study in Assendabo, southern Ethiopia, observed markedly lower diversity (*H*_E_ = 0.20) [20]. Another marker that we considered as a potential confounder of population diversity and/or structure is the msp1f3 locus. The product of the msp1f3 locus is expressed on the merozoite surface and patterns of diversity at this locus may therefore be affected by balancing selection. However, a previous study conducted on *P*. *vivax* isolates from Papua New Guinea and the Solomon Islands demonstrated that there was no evidence of balancing selection at the msp1f3 locus [24], We therefore retained MS16 and msp1f3 in our analyses but conducted additional analyses on a reduced data set without these two markers in consideration of the potential impacts of excess diversity and/or selective pressures.

A total of 197 isolates could be included in the population genetic analysis, comprising 181 (92%) isolates with complete genotype data across all 8 loci, 13 (7%) with successful calls at 7 of these loci, 2 (1%) with successful calls at 6 loci, and a single sample with successful calls at 5/8 loci. Genotyping data for the individual isolates is presented in the Supplementary Material (S4 Table). Selection bias was assessed between all enrolled participants (*n* = 353) and participants whose samples were included in the population genetic analysis (*n* = 197); no significant differences were observed in patient age, gender or peripheral parasitaemia at any of the districts (all *P* > 0.05).

### Infection Complexity and Population Diversity

A summary of polyclonality, MOI and population diversity is presented in Table 2. Moderate variation was observed in the proportion of polyclonal infections, ranging from 8% in Badawacho to 67% in Hawassa. In accordance with the polyclonality rates, the mean MOI was lowest in Badawacho (1.09) and highest in Hawassa (1.80). With the exception of Badawacho versus Halaba, all pairwise comparisons of the proportions of polyclonal infections and of the mean MOI demonstrated significant differences (*P* < 0.05). MOI did no differ significantly with either peripheral parasitemia (*rho* = 0.04, *P* = 0.558) or patient age (*rho* = 0.12, *P* = 0.100). In contrast to the complexity of infection, other than Badawacho (*H*_E_ = 0.70), *P*. *vivax* population diversity was consistently high across the sites (all *H*_E_ = 0.83). As detailed in the Supplementary Material (S5 Table), exclusion of the MS16 and msp1f3 loci did not have any notable impact on the polyclonality, MOI or population diversity in any of the study sites.

**Table 2:** Complexity of infection and population diversity.

Site	% polyclonal infections (no. polyclonal/ total no.)	Mean MOI, median (range)	Mean *H*_E_ ± SE (range)	No. of unique MLGs
Arbaminch	44% (16/36)	1.53, 1 (1–4)	0.83 ± 0.01 (0.69–0.94)	35
Halaba	21% (10/47)	1.21, 1 (1–2)	0.83 ± 0.01 (0.75–0.92)	43
Badawacho	8% (5/59)	1.09, 1 (1–2)	0.70 ± 0.01 (0.60–0.79)	25
Hawassa	67% (37/55)	1.80, 2 (1–3)	0.83 ± 0.01 (0.67–0.90)	45
All sites	35% (68/197)	1.40, 1 (1–4)	0.82 ± 0.01 (0.69–0.90)	148

### Relatedness

**Fig 2:**
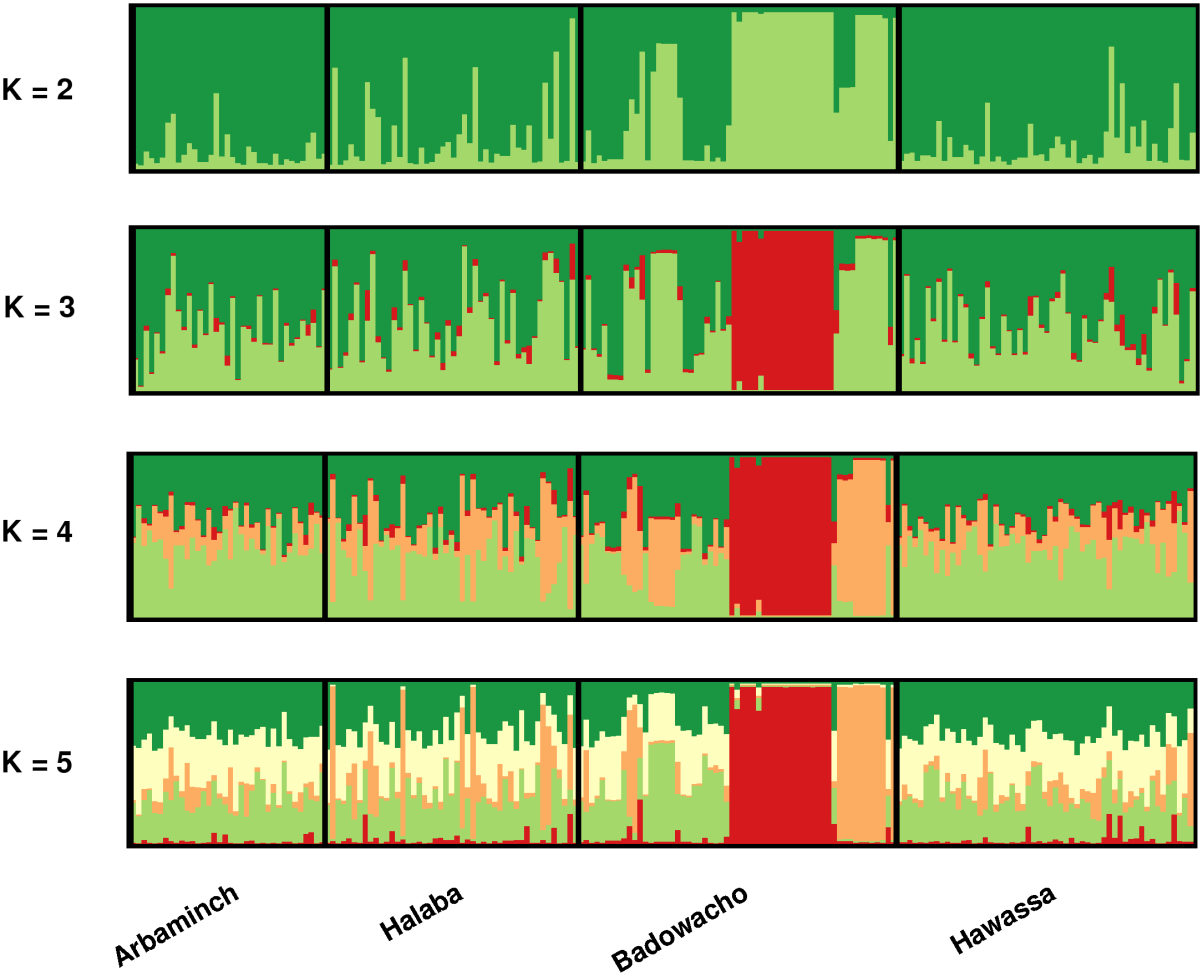
Unrooted neighbour-joining tree illustrating the genetic relatedness between the *P*. *vivax* isolates.

Neighbour-joining analysis of 181 isolates with no missing genotype data across the 8 loci highlighted clusters of isolates with 4 or more identical or near-identical multi-locus genotypes (MLGs) in Badawacho (Fig 2). The largest cluster (cluster 1) comprised 17 clonal infections with identical MLGs. A second cluster comprised two separate groups of related isolates, one with 4 identical MLGs (cluster 2a) and a second with 5 identical MLGs (cluster 2b). The isolates from the other sites exhibited overall lower genetic relatedness, with no more than two infections displaying identical MLGs.

All of the 17 isolates within cluster 1 were collected within two days of one another. A significant correlation was observed between the distance in sampling date and the proportion of alleles shared between infections in Badawacho (Mantel *r*-test, *r* = 0.52, *P* = 1.0 x 10^−5^). The correlations observed in the other sites were markedly lower, ranging from *r* = 0.017 in Arbaminch to *r* = 0.070 in Hawassa. In these three sites, only the correlation in Hawassa reached significance (*P* = 0.036).

### Linkage Disequilibrium

LD levels varied markedly between the sites, ranging from *I*_A_^S^ = 0.006 in Hawassa to *I*_A_^S^ = 0.322 in Badawacho (*P* < 0.05 in all sites except Halaba) (Table 3). However comparable levels of LD were observed in all sites when the sample set was either restricted to monoclonal infections or after accounting for isolates with identical MLGs. The only exception being in Badawacho, where the LD level declined from *I*_A_^S^ = 0.322 to 0.058 after adjusting for the repeated MLGs. Exclusion of the MS16 and msp1f3 loci did not have any notable impact on the patterns of LD in the study sites as detailed in the Supplementary Material (S6 Table).

**Table 3:** Linkage disequilibrium.

Site	All infections ^1^		Low complexity ^2^		Unique MLGs ^3^	
	*N*	*I*_A_^S^	*N*	*I*_A_^S^	*N*	*I*_A_^S^
Arbaminch	35	0.020*	25 (71%)	0.026*	35 (100%)	0.020*
Halaba	44	0.009 ^NS^	38 (86%)	0.003 ^NS^	43 (98%)	0.006 ^NS^
Badawacho	56	0.322*	53 (95%)	0.331*	25 (45%)	0.058*
Hawassa	46	0.006*	31 (67%)	0.015 ^NS^	45 (98%)	4.0 x 10^−4 NS^
All sites	181	0.051*	147 (81%)	0.074*	148 (82%)	7.0 x 10^−4 NS^

^**1**^Only samples with no missing data at all 8 loci are included in the analyses.

^2^ Restricted multi-locus haplotypes from samples with no more than one multi-allelic locus.

^3^ Unique set of multi-locus genotypes.

^NS^ Not significant (*P* > 0.05).

* *P* < 0.05.

### Population Structure and Differentiation

Population differentiation was low (*F*_ST_ < 0.2) amongst the study sites (*F*_ST_ range: 0.001–0.1) (Table 4). The highest levels of genetic differentiation were observed in comparisons against Badawacho (*F*_ST_ range: 0.065–0.1). After adjusting for the high diversity of the markers using the standardised fixation index (*F’*_ST_), differentiation remained low amongst the study sites except in comparisons against Badawacho, where the *F’*_ST_ ranged from 0.267 to 0.410 (Table 4). Furthermore, there was no marked difference in either the *F*_ST_ or *F’*_ST_ results between the full (8 marker) data set and the reduced data with exclusion of MS16 and msp1f3 (Supplementary Material; S7 Table).

**Table 4:** Pair-wise differentiation between sites.

Site	Arbaminch	Halaba	Badawacho	Hawassa
**Arbaminch**	-	0.005	0.410	0.031
**Halaba**	0.001 (*P* = 0.396)	-	0.267	0.031
**Badawacho**	0.100 (*P* <1 x10^-5^)	0.065 (*P* <1 x10^-5^)	-	0.327
**Hawassa**	0.006 (*P* = 0.117)	0.005 (*P* = 0.108)	0.079 (*P* <1 x10^-5^)	-

***F***_**ST**_ (*P-value*) in lower left triangle. ***F’***_**ST**_ in upper right triangle.

**Fig 3:**
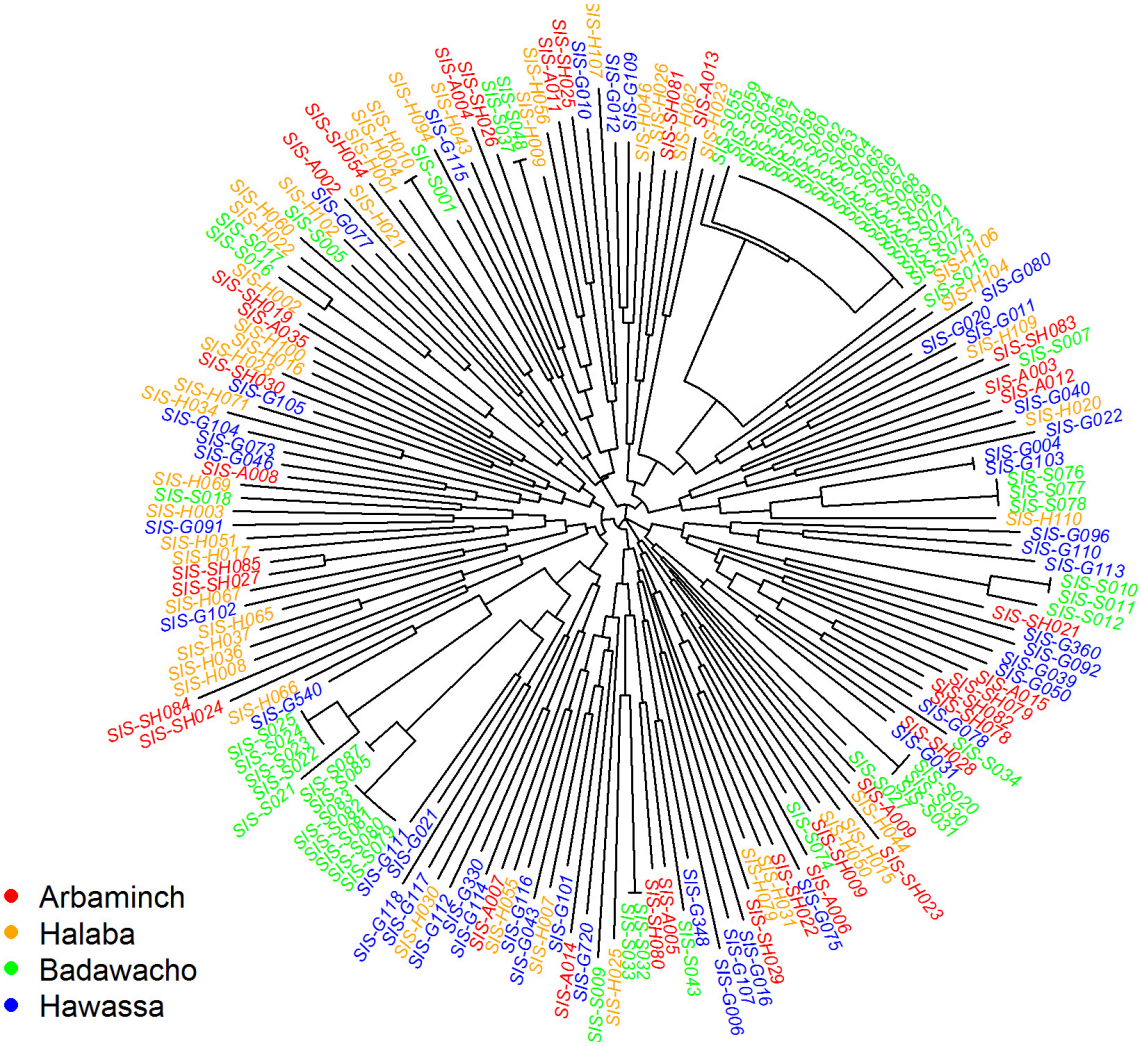
Population structure. Bar plots illustrating the population structure at *K* = 2, *K* = 3, *K* = 4 and *K* = 5. Each vertical bar represents an individual sample and each colour represents one of the *K* clusters (sub-populations) defined by STRUCTURE. For each sample, the predicted ancestry to each of the K sub-populations is represented by the colour-coded bars. *K*1 = dark green, *K*2 = light green, *K*3 = red, *K*4 = orange, and *K*5 = white.

Analysis using STRUCTURE software revealed evidence of sub-structure in the *P*. *vivax* population, largely driven by the clusters of identical isolates in Badawacho. The delta K method identified the most likely number of populations within the dataset as two (i.e. *K* = 2) (S1 Fig). At *K* = 2, the isolates were largely divided by the clusters of identical isolates (*K2*) and all remaining isolates (*K1*) (Fig 3). At *K* = 3, the largest cluster of identical isolates was clearly resolved (*K*3) and at *K* = 4, a second cluster of identical isolates was resolved (*K*4). At higher estimates of *K*, no substantial changes were observed in the population structure.

## Discussion

In the first survey of local trends in the genetic make-up of *P*. *vivax* in southern Ethiopia, significant variation was observed in population genetic parameters reflecting underlying differences in transmission between neighbouring populations. The insights derived from the genetic dynamics of the parasite population complement the traditional epidemiological surveillance approaches, and highlight a potential role for molecular surveillance in guiding evidence-based transmission intervention strategies.

One of the major differences observed amongst the districts was in the complexity of *P*. *vivax* infection. The proportions of polyclonal infections observed across the sites ranged from levels comparable to low endemic settings in Central China, Malaysia and pre-elimination regions of the Solomon Islands [15, 19, 25] (8% polyclonal infections in Badawacho), to higher transmission settings in Papua New Guinea and southeast Asia [20, 24, 28, 55] (67% polyclonal infections in Hawassa). In a previous study conducted at multiple sites in Indonesia, we observed a positive correlation between the rate of polyclonal infections and annual parasite incidence (API) indicating that polyclonality might provide a complementary gauge of local transmission intensity [55]. However, we did not observe the same trend in this study. The highest level of polyclonality (67%) was observed in Hawassa, which is home to one of the largest towns in the SNNPR: indeed Hawassa represented the most urban setting of the four sites investigated, exhibiting the second lowest *P*. *vivax* API (35 cases per 1,000 persons). It is possible that the high rates of polyclonal infection despite the low incidence in Hawassa might reflect the acquisition of malaria from satellite towns with higher *P*. *vivax* incidence on the outskirts of the main urban center. This pattern of malaria acquisition has previously been described in a study in Senegal, where individuals presented at health centers in Dakar having acquired ‘weekend malaria’ from small satellite towns surrounding the city [56]. Indeed, this trend might also explain the relatively older age of the patients from Hawassa (median 20 years) relative to the other sites (median age range from 6 to 14 years). With ever-increasing urbanization in malaria-endemic regions across the globe, further investigations will be needed to better understand the epidemiology of *P*. *vivax* in these settings. At the other end of the spectrum, the lowest rate of polyclonal infections was observed in Badawacho (8%), which represents a moderately rural setting and which exhibited the second highest *P*. *vivax* API amongst the study sites (41 cases per 1,000 persons). The low rate of polyclonal infections at this site likely reflected the unstable transmission dynamics described below. In addition to transmission patterns, patient and/or parasitological features might also influence estimates of infection complexity: however, we did not find any evidence of correlation between the proportion of polyclonal infections with patient age or parasite density within any of our study sites. Further investigations of the relationship between infection complexity and patient and parasitological details such as age, ethnicity and parasite density will require larger sample sizes in a broader range of endemic settings.

Another notable population genetic feature was the observation of large, distinct clusters of isolates with identical or near-identical multi-locus genotypes (MLGs) in Badawacho. The clonal nature of the clustered isolates, marked decline in population-level LD after accounting for repeated MLGs, and temporal clustering of identical strains, were all indicative of unstable transmission dynamics. Reaching a maximum elevation of 1,985 meters above sea level, the climate in Badawacho is especially amenable to unstable malaria transmission. Indeed, malaria had previously been eliminated from Badawacho in the 1960s but resurged several decades later, after the change of government in 1991 when populations who had been resettled in the malaria-endemic Gambella and Metekel zones returned to Badawacho [57]. After the re-introduction of malaria into the district, a number of epidemic outbreaks were observed [57], likely affecting the non-immune host population who had resided in Badawacho throughout the resettlement events.

It remains unclear what specific mechanism(s) enabled the Badawacho isolates observed in the current study to expand rapidly, particularly in the case of cluster 1, which comprised 17 strains with identical MLGs. Inspection of the clinical records including the location of patients’ homes did not reveal any evidence that the patients within any of the clusters were relatives or resided in the same households. The standing genetic diversity in Badawacho and the neighbouring districts was very high (mean *H*_*E*_ range from 0.7–0.8), hence the region could be harbouring multiple existing strains with enhanced transmission potential or drug resistance variants capable of rapid expansion under the right environmental /selective conditions. The patients from Badawacho were recruited from a clinical trial to determine the clinical efficacy of chloroquine against *P*. *vivax* (*manuscript under review*). However, 88.2% (15/17) of the infections in cluster 1 and 90% (9/10) of the infections in cluster 2 (a plus b) were cleared by day 28. Hence, the available evidence suggests that chloroquine resistance alone was not responsible for the successful expansion of the strains in clusters 1 and 2.

In Ethiopia, the health facilities are responsible for the surveillance of malaria outbreaks by means of monitoring clinical cases in healthcare facilities. However, in some regions, as many as 80% of patients with fever do not attend a health facility, and hence malaria outbreaks are often detected late after a high mortality and socio-economic burden has already been inflicted [13, 58]. Parasite genotyping may offer a complementary tool to existing malaria surveillance tools by detecting rapidly emerging strains associated that occur in the evolution of an epidemic, allowing local malaria control programs to implement appropriate interventions before the problem escalates. Prospective evaluation of the standard temporal dynamics in the local parasite genetic architecture will help to refine molecular surveillance to identify unusual and high-risk changes in the parasite population.

With the exception of Badawacho, the genetic differentiation between the study sites was generally low even after adjusting for the extensive marker diversity (*F*’_ST_ range: 0.005–0031). The higher differentiation observed in pairwise comparisons against Badawacho (*F*’_ST_ range: 0.267–0.410) was likely inflated by the sampling of multiple repeated MLGs in the ‘outbreak’ clusters. The low differentiation suggests that parasites are readily spreading from one district to another. Such dynamics have important implications for the risks of drug resistance spread, and warranting diligent surveillance of local treatment efficacy.

As demonstrated in other endemic locations [18–22, 24, 25, 33, 37, 38, 40], *P*. *vivax* population diversity was high in all the sites in the current analysis (*H*_E_ = 0.70–0.83), with no apparent correlation with transmission intensity. The factor(s) responsible for this diversity remain unclear, but may include high recombination rates supported by the contribution of relapsing infections to the overall complexity of infection, and high rates of parasite gene flow within and across sites [28, 55]. The high levels of parasite diversity facilitates the parasite adaptation in response to drug pressure and changes in environmental conditions. The extensive population diversity ensures that individual *P*. *vivax* strains can be uniquely bar-coded or finger-printed with a small and cost-effective subset of markers [59], providing a viable means for high-throughput molecular surveillance to inform local transmission dynamics.

## Supporting Information

S1 Fig Delta K method: *ΔK* against *K*. (TIFF)

S1 Table Site details. ^1^ Population estimate from 2012 based on data provided by the respective district and city administration health departments. Annual parasite incidence (API) in 2012 expressed as the number of reported cases per 1,000 population of the district(s) represented. Details on the number of reported cases in 2012 were provided by the respective district and city administration health departments.
(DOCX)

S2 Table Complexity of infection and population diversity in *P*. *vivax* isolates from Arbaminch Hospital versus Shele Health Center (Arbaminch). (DOCX)

S3 Table Marker diversity. (DOCX)

S4 Table Individual *P*. *vivax* genotypes. (XLSX)

S5 Table Complexity of infection and population diversity: comparison with and without MS16 and msp1f3. (DOCX)

S6 Table Linkage disequilibrium: comparison with and without MS16 plus msp1f3. (DOCX)

S7 Table Pair-wise differentiation between sites: comparison with and without MS16 plus msp1f3. (DOCX)
