# Supplementary figures and images for: Variation in Complexity of Infection and Transmission Stability between Neighbouring Populations of Plasmodium vivax in Southern Ethiopia
Source: PLoS One. 2015 Oct 15;10(10):e0140780. doi: 10.1371/journal.pone.0140780 (PMC4607408; doi:10.1371/journal.pone.0140780)

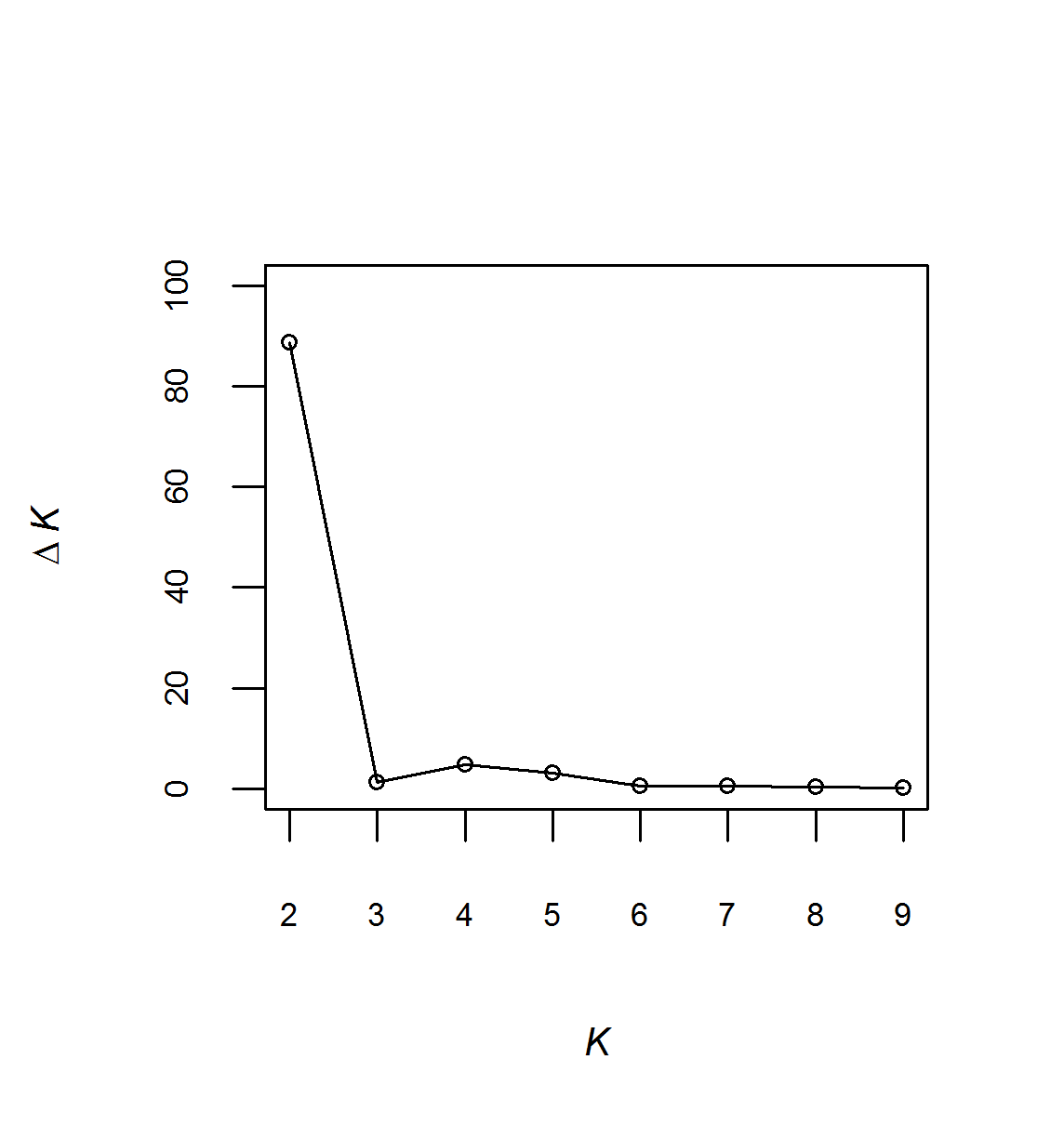

Supplement: S1 Fig — (TIFF) [file pone.0140780.s001.tiff]
