# Supplementary material for: Variation in Complexity of Infection and Transmission Stability between Neighbouring Populations of Plasmodium vivax in Southern Ethiopia
Source: PLoS One. 2015 Oct 15;10(10):e0140780. doi: 10.1371/journal.pone.0140780 (PMC4607408; doi:10.1371/journal.pone.0140780)
Supplement: S1 Table — 1 Population estimate from 2012 based on data provided by the respective district and city administration health departments. Annual parasite incidence (API) in 2012 expressed as the number of reported cases per 1,000 population of the district(s) represented. Details on the number of reported cases in 2012 were provided by the respective district and city administration health departments. (DOCX) [file pone.0140780.s002.docx]

**Table S1. Site details**

| **Site** | **Woreda (district)** | **Area, km^2^** | **Elevation** | **Population Size ^1^** | ***P. vivax* API ^2^** | **Proportion of malaria cases with *P. vivax* ^3^** |
| --- | --- | --- | --- | --- | --- | --- |
| Arbaminch | Arba Minch (town) and Zuria | 1,682 | 1,325 | 288,405 | 20.3 | 41.1% |
| Halaba | Halaba Special Woreda | 995 | 1,800 | 268,023 | 81.8 | 54.5% |
| Badawacho | Misrak Badawacho | 517 | 1,985 | 204,256 | 41.0 | 41.0% |
| Hawassa | Hawasa town | 55 | 1,708 | 328,283 | 35.4 | 28.3% |

^1^ Population estimate from 2012 based on data provided by the respective district and city administration health departments. Annual parasite incidence (API) in 2012 expressed as the number of reported cases per 1,000 population of the district(s) represented. Details on the number of reported cases in 2012 were provided by the respective district and city administration health departments.
