# Supplementary material for: Variation in Complexity of Infection and Transmission Stability between Neighbouring Populations of Plasmodium vivax in Southern Ethiopia
Source: PLoS One. 2015 Oct 15;10(10):e0140780. doi: 10.1371/journal.pone.0140780 (PMC4607408; doi:10.1371/journal.pone.0140780)
Supplement: S2 Table — (DOCX) [file pone.0140780.s003.docx]

**Table S2. Complexity of infection and population diversity in Arbaminch Hospital versus Shele Health Center (Arbaminch)**

| **Health Facility** | **No. samples** | **% Polyclonal infections** | **MOI**  **(mean, median)** | **Population diversity (mean *H*_E_)** |
| --- | --- | --- | --- | --- |
| Arbaminch Hospital | 15 | 33% (5/15) | 1.4, 1 (1-3) | 0.825 |
| Shele Health Center | 21 | 52% (11/21) | 1.62, 1 (1-4) | 0.823 |
|  | 36 | *P* = 0.427 | *P* = 0.324 | *P* = 0.958 |
