# Supplementary material for: Variation in Complexity of Infection and Transmission Stability between Neighbouring Populations of Plasmodium vivax in Southern Ethiopia
Source: PLoS One. 2015 Oct 15;10(10):e0140780. doi: 10.1371/journal.pone.0140780 (PMC4607408; doi:10.1371/journal.pone.0140780)
Supplement: S3 Table — (DOCX) [file pone.0140780.s004.docx]

**Table S3. Marker diversity**

| **Marker** | **Arbaminch** | **Halaba** | **Badowacho** | **Hawassa** | **All districts** |
| --- | --- | --- | --- | --- | --- |
| MS20 | 0.94 | 0.92 | 0.78 | 0.88 | 0.90 |
| Pv3.27 | 0.87 | 0.86 | 0.79 | 0.90 | 0.88 |
| MS10 | 0.87 | 0.82 | 0.79 | 0.85 | 0.85 |
| MS1 | 0.86 | 0.84 | 0.69 | 0.83 | 0.83 |
| MS16 | 0.77 | 0.82 | 0.69 | 0.87 | 0.82 |
| MS12 | 0.83 | 0.83 | 0.62 | 0.85 | 0.82 |
| MS5 | 0.77 | 0.75 | 0.60 | 0.78 | 0.78 |
| Msp1F3 | 0.69 | 0.75 | 0.65 | 0.67 | 0.69 |
