# Supplementary material for: Variation in Complexity of Infection and Transmission Stability between Neighbouring Populations of Plasmodium vivax in Southern Ethiopia
Source: PLoS One. 2015 Oct 15;10(10):e0140780. doi: 10.1371/journal.pone.0140780 (PMC4607408; doi:10.1371/journal.pone.0140780)
Supplement: S5 Table — (DOCX) [file pone.0140780.s006.docx]

**Table S5. Complexity of infection and population diversity: comparison with and without MS16 and msp1f3**

|  | **% polyclonal infections (no. polyclonal/total no.)** | | **Mean MOI, median (range)** | | **Mean *H*_E_ ± SE (range)** | | **No. of unique MLGs** | |
| --- | --- | --- | --- | --- | --- | --- | --- | --- |
| **Site** | **8 Markers** | ***6 Markers** | **8 Markers** | ***6 Markers** | **8 Markers** | ***6 Markers** | **8 Markers** | ***6 Markers** |
| Arbaminch | 44% (16/36) | 42% (15/36) | 1.53, 1 (1-4) | 1.50, 1 (1-4) | 0.83 ± 0.01 (0.69 – 0.94) | 0.86 ± 0.02 (0.77 – 0.94) | 35 | 34 |
| Halaba | 21% (10/47) | 21% (10/47) | 1.21, 1 (1-2) | 1.21, 1 (1-2) | 0.83 ± 0.01 (0.75 – 0.92) | 0.84 ± 0.02 (0.75 – 0.92) | 43 | 43 |
| Badawacho | 8% (5/59) | 8% (5/59) | 1.09, 1 (1-2) | 1.08, 1 (1-2) | 0.70 ± 0.01 (0.60 – 0.79) | 0.71 ± 0.04 (0.60 – 0.79) | 25 | 24 |
| Hawassa | 67% (37/55) | 64% (35/55) | 1.80, 2 (1-3) | 1.75, 2 (1-3) | 0.83 ± 0.01 (0.67 – 0.90) | 0.84 ± 0.02 (0.78 – 0.90) | 45 | 45 |
| All sites | 35% (68/197) | 33% (65/197) | 1.40, 1 (1-4) | 1.38, 1 (1-4) | 0.82 ± 0.01 (0.69 – 0.90) | 0.84 ± 0.02 (0.73 – 0.90) | 148 | 146 |

*Excluding MS16 and msp1f3
