# Supplementary material for: Variation in Complexity of Infection and Transmission Stability between Neighbouring Populations of Plasmodium vivax in Southern Ethiopia
Source: PLoS One. 2015 Oct 15;10(10):e0140780. doi: 10.1371/journal.pone.0140780 (PMC4607408; doi:10.1371/journal.pone.0140780)
Supplement: S6 Table — (DOCX) [file pone.0140780.s007.docx]

**Table S6. Linkage disequilibrium: comparison with and without MS16 plus msp1f3**

|  | ***I*_A_^S^ All infections ^1^  (*n*)** | | ***I*_A_^S^ Low complexity ^2^ (*n*)** | | ***I*_A_^S^ Unique MLGs ^3^ (*n*)** | |
| --- | --- | --- | --- | --- | --- | --- |
| **Site** | **8 Markers** | **6 Markers** | **8 Markers** | **6 Markers** | **8 Markers** | **6 Markers** |
| Arbaminch | 0.020* (35) | 0.021* (35) | 0.026* (25) | 0.024* (27) | 0.020* (35) | 0.013 ^NS^ (34) |
| Halaba | 0.009 ^NS^ (44) | 0.004 ^NS^ (44) | 0.003 ^NS^ (38) | -0.004 ^NS^ (38) | 0.006 ^NS^ (43) | -0.002 ^NS^ (43) |
| Badawacho | 0.322* (56) | 0.318* (57) | 0.331* (53) | 0.339* (54) | 0.058* (25) | 0.041* (25) |
| Hawassa | 0.006* (46) | 0.015 ^NS^ (47) | 0.015 ^NS^ (31) | 0.018 ^NS^ (34) | 4.0 x 10^-4^ ^NS^ (45) | 0.009 ^NS^ (46) |
| All sites | 0.051* (181) | 0.055* (183) | 0.074* (147) | 0.076* (153) | 7.0 x 10^-4^ ^NS^ (148) | -0.002 ^NS^ (148) |

**^1^**Only samples with no missing data at all loci are included in the analyses.

^2^ Restricted multi-locus haplotypes from samples with no more than one multi-allelic locus.

^3^ Unique set of multi-locus genotypes.

^NS^ Not significant (*P* > 0.05).

* *P* < 0.05.
