# Supplementary material for: Variation in Complexity of Infection and Transmission Stability between Neighbouring Populations of Plasmodium vivax in Southern Ethiopia
Source: PLoS One. 2015 Oct 15;10(10):e0140780. doi: 10.1371/journal.pone.0140780 (PMC4607408; doi:10.1371/journal.pone.0140780)
Supplement: S7 Table — (DOCX) [file pone.0140780.s008.docx]

**Table S7. Pair-wise differentiation between sites: comparison with and without MS16 plus msp1f3**

| **Site** | **Marker set** | **Arbaminch** | **Halaba** | **Badawacho** | **Hawassa** |
| --- | --- | --- | --- | --- | --- |
| **Arbaminch** | **8 markers** | - | 0.005 | 0.410 | 0.031 |
|  | ***6 markers** | - | 0.028 | 0.451 | 0.033 |
| **Halaba** | **8 markers** | 0.001 (*P* = 0.396) | - | 0.267 | 0.031 |
|  | ***6 markers** | 0.004 (*P* = 0.225) | - | 0.276 | 0.037 |
| **Badawacho** | **8 markers** | 0.100 (*P* <1 x10^-5^) | 0.065 (*P* <1 x10^-5^) | - | 0.327 |
|  | ***6 markers** | 0.100 (*P* <1 x10^-5^) | 0.063 (*P* <1 x10^-5^) | - | 0.359 |
| **Hawassa** | **8 markers** | 0.006 (*P* <1 x10^-5^) | 0.005 (*P* = 0.108) | 0.079 (*P* <1 x10^-5^) | - |
|  | ***6 markers** | 0.005 (*P* = 0.207) | 0.006 (*P* = 0.006) | 0.081 (*P* <1 x10^-5^) | - |

***F*_ST_** (*P-value*) in lower left triangle. ***F’*_ST_** in upper right triangle.

*Without MS16 and msp1f3
